# Temperature-controlled laser thermal therapy system using a newly developed laparoscopic system equipped with an ultra-compact thermographic camera

**DOI:** 10.1038/s41598-022-22908-4

**Published:** 2022-10-31

**Authors:** Manabu Harada, Yuji Morimoto, Ohara Mutsuki, Jun Ohya, Ken Masamune, Yujiro Itazaki, Takao Sugihara, Hironori Tsujimoto, Yoji Kishi, Hideki Ueno

**Affiliations:** 1 Department of Surgery, National Defense Medical College, Saitama, Japan; 2 Department of Physiology, National Defense Medical College, Namiki 3-2, Tokorozawa, Saitama 359-8513 Japan; 3 Department of Modern Mechanical Engineering, School of Creative Science and Engineering, Waseda University, Tokyo, Japan; 4 Faculty of Advanced Techno-Surgery, Institute of Advanced Biomedical Engineering and Science, Tokyo Women’s Medical University, Tokyo, Japan

**Keywords:** Translational research, Biomedical engineering

## Abstract

Laser thermal therapy is one of the treatments for malignant tumors. We developed a thermal endoscope using an ultra-compact thermo-sensor and established a new laparoscopic laser thermal therapy system to heat cancer tissue at an appropriate temperature, focusing on the fact that thermographic cameras are capable of two-dimensional temperature mapping. Hepatocellular carcinoma (N1S1) cells were implanted into the livers of Sprague–Dawley rats (n = 13) to create orthotopic hepatocellular carcinoma. Six of the rats underwent laparoscopic laser thermotherapy (70 °C, 5 min) using the newly developed system, and the others underwent laparoscopic insertion only. Lesion volume measurement and histological evaluation were performed in all of the rats. The laparoscopic laser thermal therapy system provided stable temperature control. When a temperature of 70 °C was used for the set temperature, the temperature of the target cancer was maintained within the range of 68–72 °C for 93.2% of the irradiation time (5 min). The median volume of the tumors that were thermally treated was significantly smaller than that of the untreated tumors. The newly developed laparoscopic laser thermal therapy system was capable of maintaining the temperature of the tumor surface at any desired temperature and was proven to be effective in treatment of the rat hepatocellular carcinoma model.

## Introduction

Thermal therapy is a highly effective treatment for cancer because cancer cells are vulnerable to heat, and thermal therapy has been studied for a long time because of its minimal adverse effects^1–3^.

In recent years, laser thermal therapy (LTT), a method for heating tumor tissue by laser irradiation, has attracted attention. Thermal heating by laser light occurs when light energy is absorbed by tissue and then converted to heat^4^. Laser light's absorption in tissues varies depending on the tissue's constituent components (proportions of extracellular matrix, collagen, water, etc.), each of the organs having its own characteristics^5^. However, once converted to localized heating of the tissue, the thermodynamic effect on the tissue is the same. The therapeutic effect of laserthermia is caused by tissue destruction due to vaporization of water in the tissue and apoptosis or necrosis of tumor cells^6^. Since LTT can be applied to organs inside the body by using optical fiber, LTT can be used not only for cancers of luminal organs such as the esophagus^7^ but also cancers of solid organs such as liver cancer^8^, brain tumor^9^, and renal cell carcinoma^10^.

To achieve safe and effective LTT, it is necessary to monitor the temperature of the cancer tissue during heating and to maintain the temperature at an appropriate level. Magnetic resonance imaging (MRI)-based temperature monitoring has been used in interstitial LTT for brain tumors, and the effectiveness of temperature control in treatment has been proved^9,11,12^. On the other hand, temperature monitoring based on radiant energy (infrared) detection has the advantages of obtaining the surface temperature of an object (1) non-invasively and (2) in real time. In addition, (3) the two-dimensional thermal distribution can be obtained. Based on such advantages, we have established a temperature monitoring method using a thermographic camera and have shown its usefulness in laser thermotherapy. Specifically, we succeeded in developing a feedback system that automatically controls the laser output using temperature information obtained from the thermographic camera as an input signal while heating the target tissue^13^. Using the system, we demonstrated that the temperature of the target tumor can be maintained at a stable temperature in an animal model^13^, and we reported that this leads to a good therapeutic effect^14^.

On the other hand, in recent years, laparoscopic surgery for intraperitoneal malignancies has become widely used as a minimally invasive treatment for cancer. In the laparoscopic technique, operators make use of carbon dioxide insufflation to inflate the abdominal cavity, thus leading to excellence in observation and treatment for cancers deeply located in cavities such as the pelvic cavity and under the diaphragm. Therefore, laparoscopic surgery is currently used for various cancers such as gall bladder and hepatocellular carcinoma in addition to gastric, colon, and rectal cancers^15,16^. Thus, we came up with the idea of introducing thermal therapy as an assistive treatment in laparoscopic surgery. Thermal therapy can be applied to tumors for which surgical resection is difficult (e.g., tumors with indistinct boundaries or tumors involving large blood vessels), and it can therefore compensate for shortcomings in surgical methodology.

In order to apply LTT to laparoscopic surgery, we have developed a laparoscope system equipped with a compact thermopile array sensor^17^. This laparoscope system has a laser forceps hole and a rigid endoscope in addition to the thermopile array sensor. The system can simultaneously acquire both an image of the observation site and a two-dimensional map of the surface temperature, and it enables maintenance of constant heating of the target tissue at a set temperature. In this study, to prove the usefulness of this laparoscopic treatment system, we performed non-contact LTT under laparoscopic conditions in a rat orthotopic hepatocellular carcinoma model, and we verified its therapeutic efficacy.

## Results

### Evaluation of the anti-tumor effect using the temperature-controlled LTT (TC-LTT) system

The rat model of hepatocellular carcinoma was treated with non-contact TC-LTT under laparoscopy at 70 °C for 300 s. The reason for setting the heating time to 300 s was based on the results of a previous study in which radiofrequency ablation was used for treatment of hepatocellular carcinoma^18^. The heating temperature setting (70 °C) was determined on the basis of the results of a preliminary study that was carried out to investigate the relationship between heating temperature and depth of treatment (Suppl. Fig. S1). Since the thickness of the tumor in the model animal was approximately 6 mm, 70 °C was selected as the minimum required temperature at which that thickness could be treated. This temperature setting was also intended to minimize injury to normal tissue.

**Figure 1:**
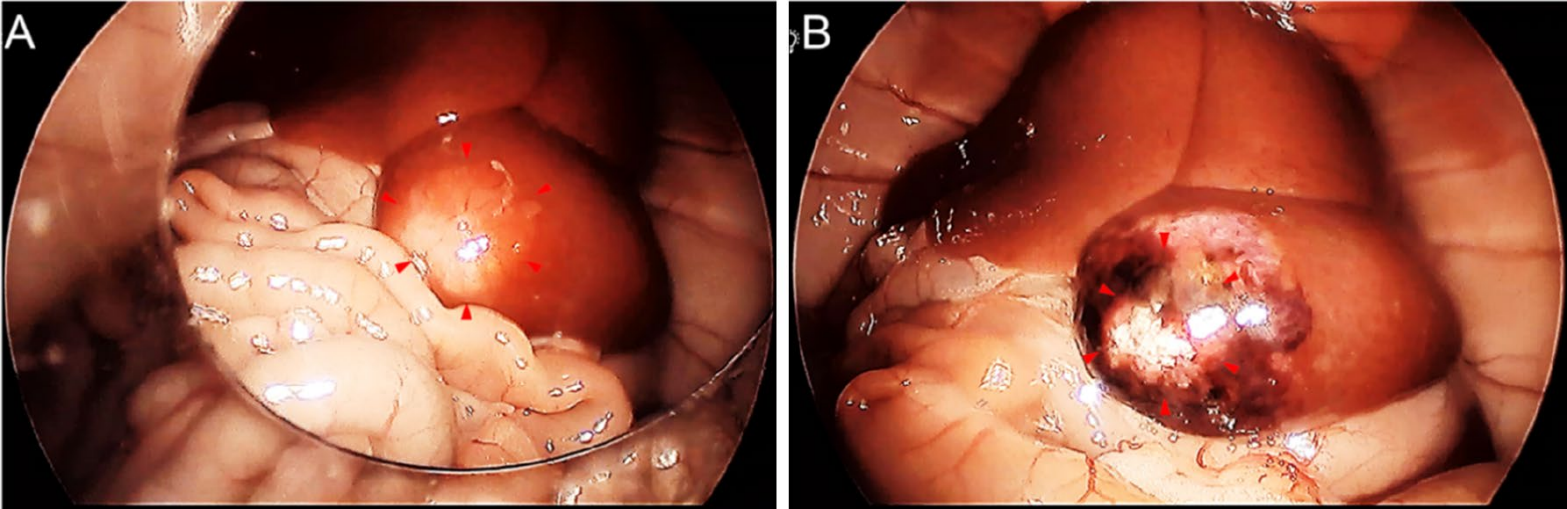
Laparoscopic views of rat liver cancer (*red triangle*) before (**A**) and after (**B**) thermal treatment.

Figure 1 shows intra-abdominal images of the rat model of orthotopic hepatocellular carcinoma observed by laparoscopy (AIM1588, Stryker, San Jose, CA, USA) before and after treatment. Hepatocellular carcinoma recognized as a white nodular lesion in the left lateral lobe before the thermal therapy (Fig. 1A) was degenerated after the thermal therapy (Fig. 1B). As shown in Fig. 1, the surface of the tumor sometimes turned black after the laser irradiation. Since the temperature was controlled at 70 °C, it is unlikely that this was carbonization, and the coloration probably originated from methemoglobin produced by heating of hemoglobin^19^ (Suppl. Sec. 4).

Supplementary Video shows the actual treatment in progress. Thermal imaging (left side of the video) showed that the irradiated area was heated and the temperature increased after the start of laser irradiation. As the surgeon fine-tuned the irradiation site, bright field imaging (right side of the video) showed that the laser irradiation site continued to overlap the tumor site. Just at the end of laser irradiation, the reflecting laser illumination at the tumor site disappeared in the bright field imaging and, at the same time, the color of the dot representing the maximum temperature in the thermal imaging changed from red to green.

**Figure 2:**
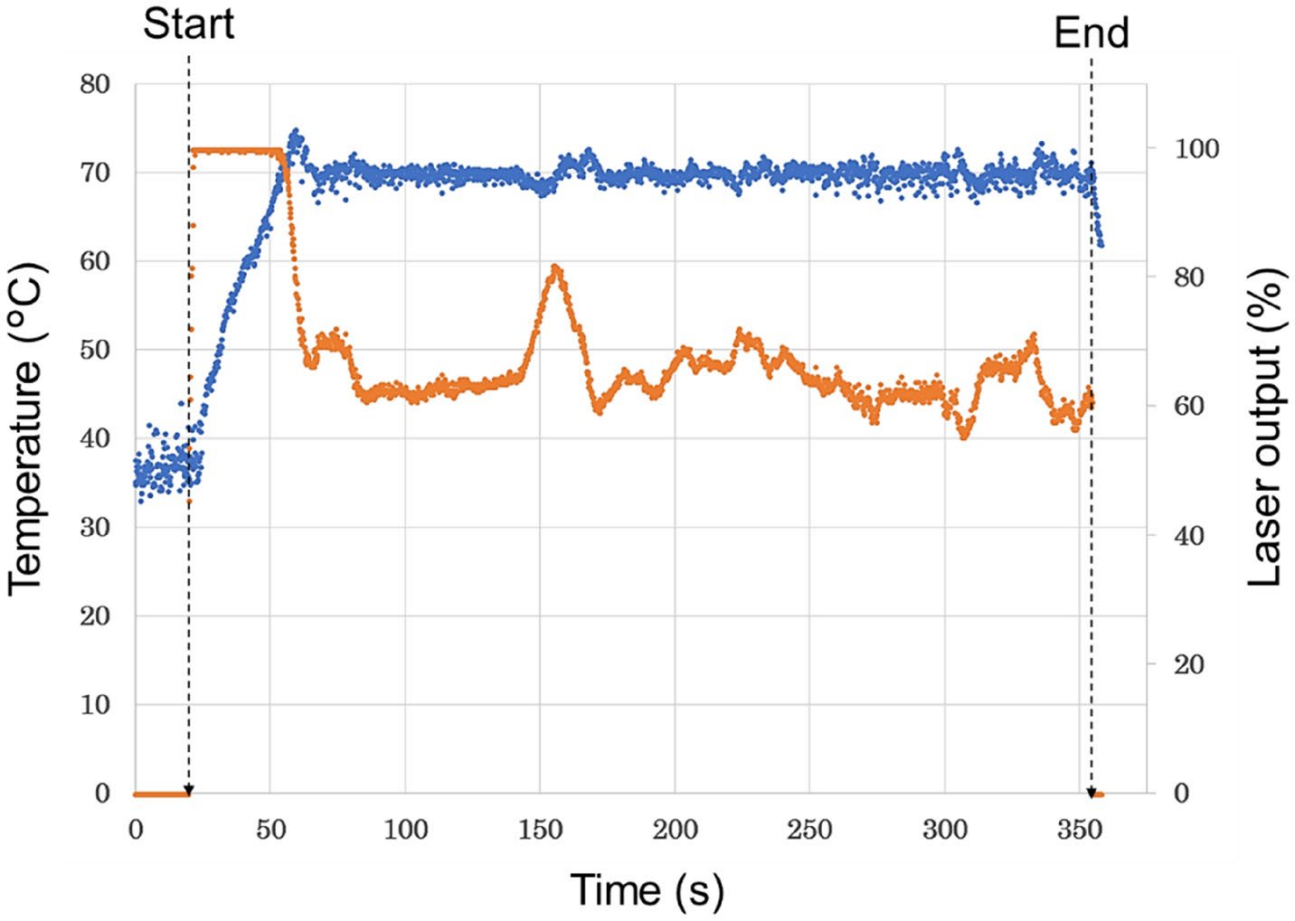
Temperature of the tumor surface *(blue dots*) and laser power (*orange dots*) over time during laser thermal therapy. The tumor surface temperature rises with the start of laser irradiation, and once the temperature reaches the set temperature (70 °C), the laser power is automatically controlled so that the tumor surface temperature is maintained at 70 °C.

**Figure 3:**
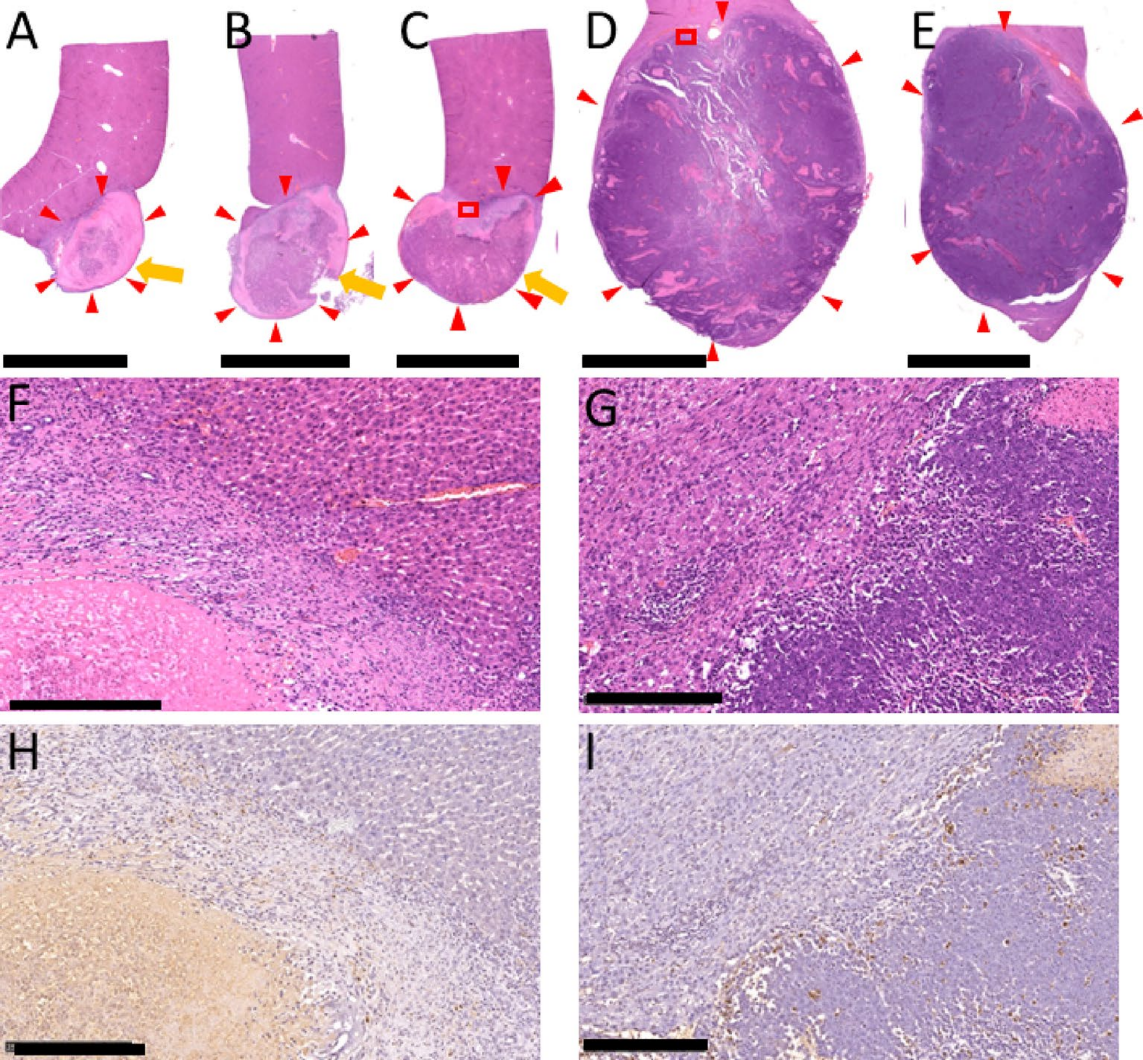
(**A**), (**B**) and (**C**), Macroscopic images of the thermally treated tumors (*red triangle*). Each image was from one of three different animals. The direction of laser irradiation is indicated by an *orange arrow*. (**D**) and (**E**), Macroscopic images of the non-treated tumors (*red triangle*). Each image was from one of two different animals. Photograph (**F**) is an enlarged view of the *red frame* in photograph (**C**), a frame placed on the boundary between the normal liver tissue and tumor tissue. Photograph (**H**) shows necrotic changes (TUNEL-positive) in the tumor. Photograph (**G**) is an enlarged view of the *red frame* in photograph (**D**), a frame placed on the boundary between the normal liver tissue and tumor tissue. Each tissue sample was cut to maximize the area on the sagittal plane of the tumor. HE, Scale bar = 5 mm (**A**, **B**, **C**, **D**), 0.25 mm (**F**, **G**), TUNEL, Scale bar = 0.25 mm (**H**, **I**).

Figure 2 shows the changes in tumor temperature and laser power value during the thermal treatment. The laser power was maximized within 1 s after the start of laser irradiation, and the tumor temperature reached the set temperature (70 °C) after about 30 s of irradiation at the maximum power. The maximum laser power for the apparatus was 3 W/cm^2^. It can be confirmed that the laser power was automatically controlled to keep the tumor surface temperature constant at 70 °C for the following 300 s. After the tumor temperature reached 70 °C, the median tumor temperature during temperature control was 69.8 °C (min of 67.8, max of 77.4 °C), with a distribution of temperature variation of < 68 °C: 0.2%, 68–72 °C: 93.2%, and > 72 °C: 6.6%. Hematoxylin and eosin (HE)-stained and terminal deoxynucleotidyl transferase (TdT) dUTP Nick-End Labeling (TUNEL)-stained specimens are shown in Fig. 3. In the treatment group, necrotic degeneration was observed throughout the tumor area, and normal liver tissue bordering the tumor margin was also thermally degenerated with a thickness of approximately 1.5 mm (median thickness of 1.4 mm (min of 0.6, max of 2.6 mm)).

**Figure 4:**
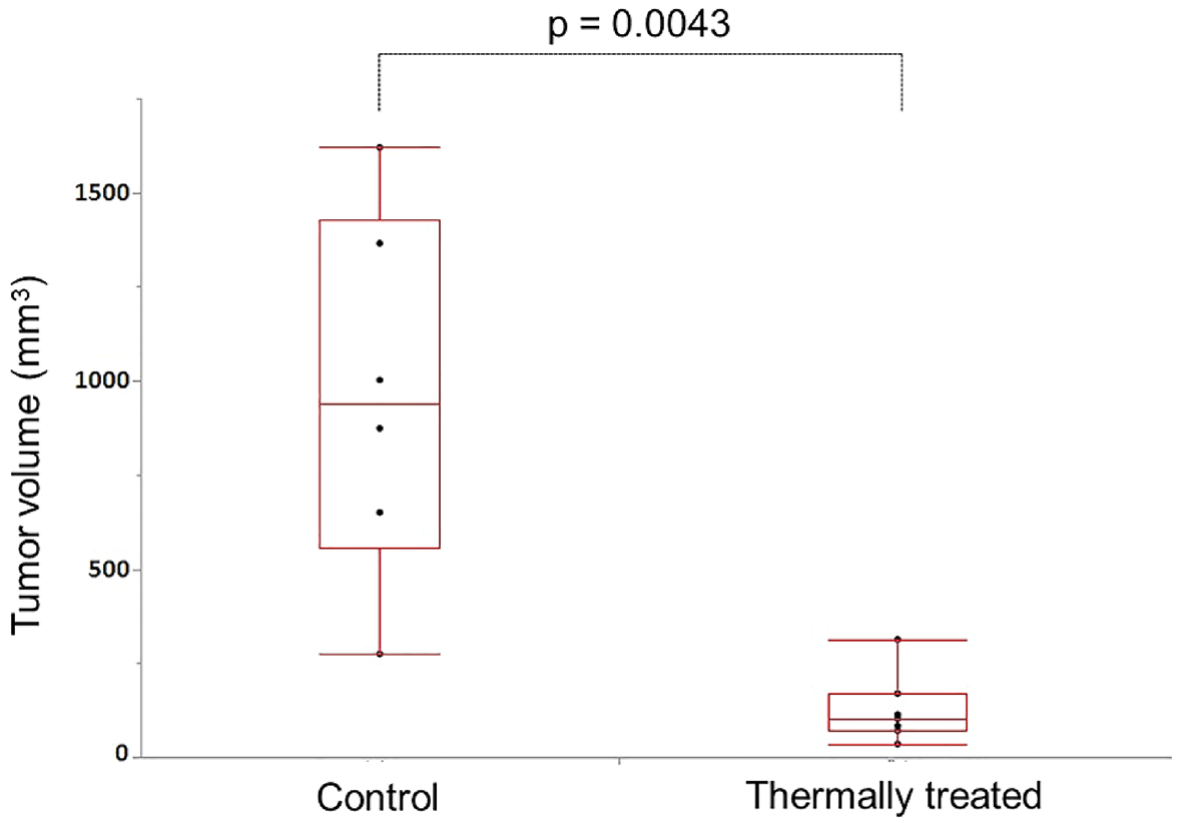
Scatter plots of individual tumor volumes in the thermally treated group and in the control group. Significant reduction of tumor volume was seen in the thermally treated group (*P* = 0.0043).

Tumors volumes at the time of sacrifice in the treatment group and control group are shown in Fig. 4. The median tumor volume was significantly smaller in the treatment group (treatment group: 1.0 × 10^2^ mm^3^, control group: 9.4 × 10^2^ mm^3^, *P* = 0.0043). The histopathological results suggest that necrosis of the entire tumor area occurred in the treatment group and that tumor growth was almost completely suppressed.

None of the mice were found to be unhealthy or dead throughout this study, and there were no treatment-related deaths in the treatment group. In addition, local abscesses and hematomas that were seen in previous studies^20,21^ were not observed.

## Discussion

In this study, we successfully eradicated cancer tissue by non-contact LTT in an orthotopic animal tumor model using the newly developed temperature-controlled laparoscopic laser thermal therapy (TC-LTT) system. Continuous monitoring with a thermo-sensor without a time lag enabled real-time imaging of the two-dimensional temperature distribution of the irradiated area. The surgeon could know in real time whether heating of the tumor was being performed without excess or deficiency. In addition, the feedback mechanism of laser power by temperature monitoring enabled precise temperature control of the target lesion during heating.

In order to achieve a good therapeutic effect in thermal therapy for malignant tumors, it is important to heat and maintain the tissue at an appropriate temperature. Our preliminary experiments showed that both low and excessively high treatment temperatures led to inadequate results (Suppl. Fig. S1). It has also been reported that overheating or underheating results in unwanted thermal effects including vaporization, char (Suppl. Fig. S3), and applicator damage or failure^22^.

Temperature monitoring during laser interstitial thermal therapy (LITT) for malignant tumors has been reported using thermocouples, MRI, computed tomography (CT), and other temperature measurement methods^22^. Temperature measurement with no time lag is possible with a thermocouple, but it requires insertion of a thermocouple into the tissue, which poses a risk of bleeding and tumor seeding. On the other hand, temperature measurement with CT or MRI is attractive because it is non-invasive and enables measurement of the temperature distribution in three dimensions (temperature resolution: ± 0.2 °C). However, the system in MRI has a time lag of 4–5 s before the measurement and cannot follow the temperature change in seconds^23^. In addition, MRI is difficult to adapt to unfixed organs due to noise caused by body movements^22^. CT has the problem of exposure of biological tissue to ionizing radiation. On the other hand, the greatest advantage of the TC-LTT system is that the temperature distribution can be obtained in almost real time (time lag of only 0.12 s) and in two dimensions non-invasively without the use of ionization radiation. In addition to the control of heating by the thermo-sensor, this system enables the surgeon to see the process associated with thermal changes in the tissue under treatment in bright field images, allowing the surgeon to perform treatment with confidence.

Since this system is a non-contact (no puncture) form of temperature measurement and laser irradiation to the target tissue, there is no mechanical invasion of the tumor. In the case of LTT of solid organs, an interstitial irradiation method in which a laser fiber is punctured into the tumor and heated is generally used. However, puncture operations on tumors pose the risk of bleeding and puncture-related tumor seeding^24^. In addition, puncture-type light-emitting devices (e.g., optical laser probes of the NeuroBlate system (Monteris Medical, MN, USA)) generally require a cooling system to prevent overheating of the probe tip, which not only complicates the operation but also poses a risk of physical injury due to breakage. On the other hand, our established TC-LTT system uses bare fibers in a non-puncture manner, which eliminates the above risks.

LTT for patients with early-stage hepatocellular carcinoma has fewer complications and is as effective as surgery in the short term^24^.

When the tumor tissue is heated to a temperature in the range of 50 °C to 100 °C, it causes coagulative necrosis^25^. However, heating above 100 °C poses the risk of tumor rupture, carbonization and incomplete coagulation necrosis due to vaporization of water in the tissue. Therefore, it is desirable to supply heat energy of between 50 and 100 °C to the entire tumor area in order to completely treat the tumor. Temperature control is necessary to avoid side effects. Even at the laser power set in this study (3 W/cm^2^), without temperature control, the tumor surface temperature exceeded 100 °C, resulting in carbonization (Suppl. Fig. S3).

If a high-power laser is used, the temperature of the irradiated area may become higher than the set temperature before the temperature control mechanism is activated. However, in the setup used in this study (maximum laser power: 3 W/cm^2^), such an event was not observed, and the temperature rise during sampling time (0.12 s) was only about 0.1 °C. A rise in temperature above the set temperature during sampling in the case of using a high-power laser in the future can be avoided by (1) lowering the maximum laser power setting and (2) shortening the sampling time.

In this study, the treatment time of 300 s was based on the results of a previous study in which radiofrequency ablation was used for treatment of hepatocellular carcinoma^18^, but an additional experiment revealed that tumor growth suppression can be attained even in shorter times (Suppl. Fig. S2). In the additional experiment (Suppl. Sec. 2), a heating time of 150 s resulted in a tumor necrosis depth comparable to that with 300-s heating. However, at further shorter heating times (less than 75 s), the depth of tumor necrosis fluctuated (become unstable). Surprisingly, however, in some cases, 37-s heating induced a depth of tumor necrosis equivalent to that of 300-s heating. Therefore, it is possible that the procedure can be completed in a shorter time than 150 s if a device that can heat the entire tumor homogenously (e.g., a device for heating with slight variation in the distance between the fiber probe and tumor surface during the heating) is used.

The fluence rate was estimated as follows. First, the beam spot diameter at the irradiated surface was measured with respect to the distance from the optical fiber tip position to the irradiated surface (Suppl. Fig. S5). Since the distance between the endoscope tip and the tumor surface was estimated to be about 10 mm during the operation in the rat abdominal cavity, the estimated beam spot diameter on the tumor at that time was about 10 mm (in terms of area, 0.79 cm^2^) as shown in Suppl. Fig. S5. Therefore, the fluence rate during intraperitoneal manipulation can be estimated to be 3.8 W/cm^2^ at the laser power of 3 W/cm^2^.

The optical depth of near-infrared light in living tissue is about 5 mm^26^. However, in this study, a treatment maximum depth of up to 9.3 mm was obtained (Suppl. Fig. S1). This is probably due to heat transfer from the heated tissue rather than direct heating by absorption of the near-infrared light. Therefore, it was found that a treatment depth exceeding the optical depth could be obtained if the heating was maintained under a certain temperature.

Further miniaturization is practically possible by reducing the size of the thermopile array. In this study, a thermopile array with a spatial resolution of 32 × 32 (Φ = 9 mm) was used, resulting in an endoscope outer diameter of 14 mm. At present, 5.3 mm thermopile arrays (HTPA8 × 8d (spatial resolution of 8 × 8 pixels), Heimansensor, Germany) are commercially available, and the tip outer diameter of the endoscope could therefore be downsized to approximately 9 mm. However, it would still be difficult to reduce the outer diameter of the endoscope tip to 1–5 mm.

### Limitations

In this study, the temperature distribution in the depth direction from the irradiated area to the antipode area was unknown. However, observation of a histopathological specimen showed that the median thermal depth from the irradiated point to the antipodal point was 4.3 (min: 3.2, max: 4.7) mm, and the thermal energy reached the entire tumor area in this tumor model. In addition, there was no unexpected thermal injury to other organs as the degree of injury to normal liver tissue was small and there was no treatment-related death.

The maximum treatment depth obtained in this study was approximately 9 mm. However, taking into account the tissue penetration depth of 808 nm light^26^, the therapeutic effect on tumors thicker than that depth would be insufficient. However, many light-absorbing nano-agents with high thermal conversion efficiency have been reported^27^, and it may be possible to enhance the therapeutic effect by combining such agents.

The distance between the tumor and the optical fiber tip varied due to movement of the liver linked to diaphragmatic respiratory movements and due to motion of the laparoscope by the surgeon's handling, and the spot size was not constant. However, it was possible to continue targeting the tumor by modifying the irradiation position based on observation of bright field images and thermal images. The main factors that cause the spot size to change are displacement of the tip position due to the movement of the organ and quivering of the operator's hand. It is better to have as little variation in beam size as possible, and a possible measure to achieve this is to fix the endoscope by a machine rather than by an operator's hand. In the future, if an endoscope tip position feedback compensation system based on image tracking is constructed, it will be possible to minimize size changes due to organ movement.

As for future prospects, since LTT for cancer has been reported to be useful in other types of cancer, the thermal endoscope-based TC-LTT may be applicable to other cancer types in the future. Since the thermal endoscope-based TC-LTT system can be used in a non-contact manner for a lesion, it may be a good indication for intraepithelial lesions in the gastrointestinal tract or lesions with a high bleeding risk that are difficult to treat with endoscopic mucosal resection or endoscopic submucosal dissection^28–30^.

In conclusion, we constructed a laparoscopic TC-LTT system with a thermal endoscope equipped with an ultra-compact thermo-sensor, a complementary metal oxide semiconductor (CMOS) camera, and a channel for an optical fiber and with an automatic control system for laser output. Using this system, non-contact TC-LTT was performed laparoscopically in a rat model of orthotopic hepatocellular carcinoma and the carcinoma was successfully eradicated. The results suggest that non-contact TC-LTT can be performed laparoscopically and may be an effective treatment for cancer of solid organs.

## Methods

### TC-LTT system

**Figure 5:**
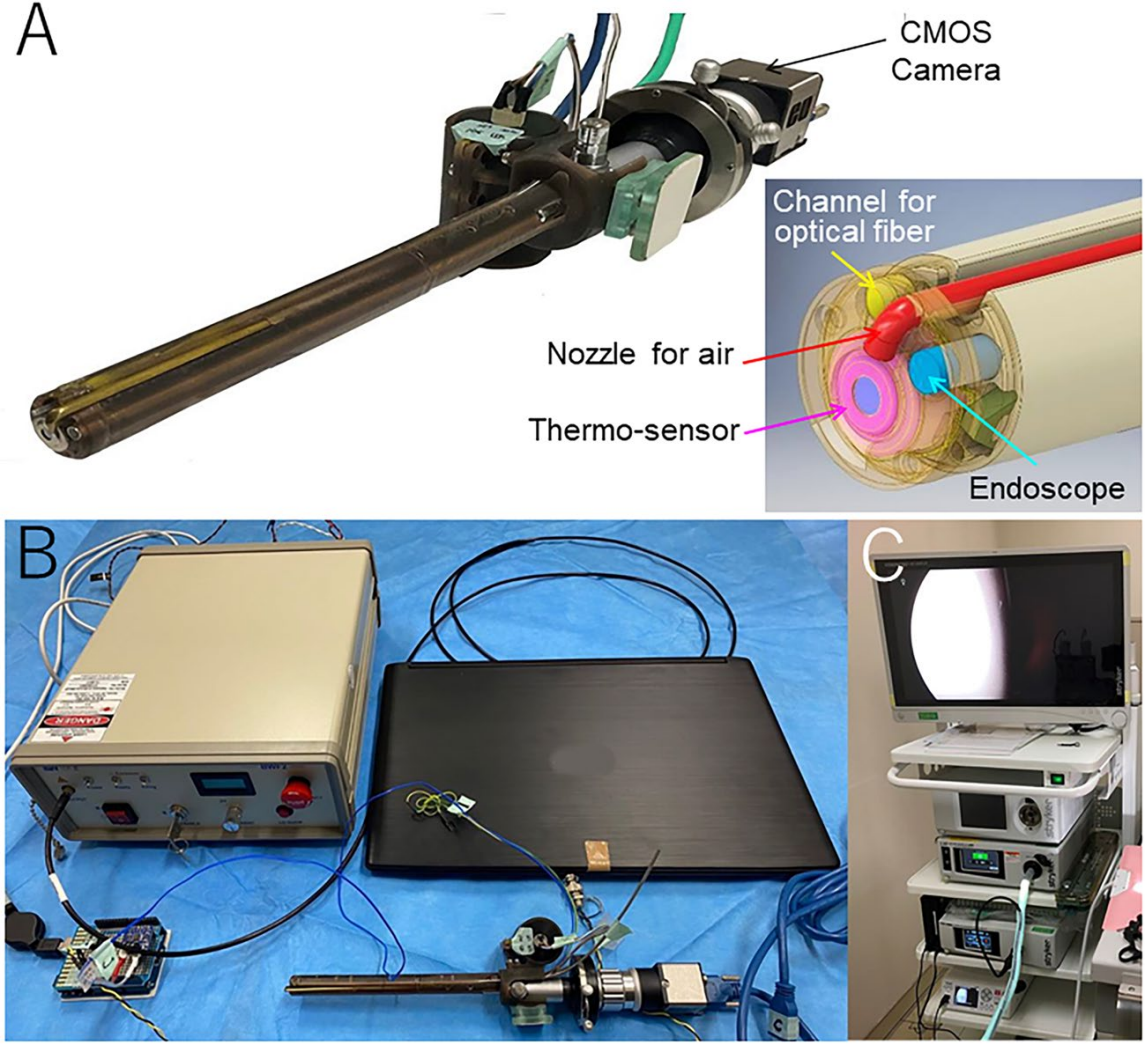
(**A**) Laparoscopic thermal camera and its bird's eye view of the tip assembly (*lower right*). The tip assembly is composed of a channel for an optical fiber, a rigid endoscope, a nozzle for air and a thermo-sensor. (**B**) Configuration of the temperature-controlled laser thermal therapy system. The system consists of a laparoscopic thermal endoscope (*lower right*), laser generator (*upper left*), control PC (*upper right*) and microcontroller (*lower left*). (**C**) Light source and insufflation system for the laparoscope.

The constructed thermal endoscope consisted of a rigid endoscope (the shaft having a maximum diameter of 14 mm and length of 288 mm) (serial No. 11499, Shinko Koki, Japan), an ultra-compact infrared thermography sensor (HTPA32 × 32d L2.1, Heimann Sensor, Germany), and a channel for introducing an optical fiber for laser irradiation^17^ (Fig. 5A).

The two-dimensional temperature distribution was visualized by the thermography sensor with a frame rate of 8.3 fps and a spatial resolution of 32 × 32 pixels (A temperature range of 20–80 °C corresponds linearly to a pixel value of 0–255.). Bright field images were obtained by a CMOS camera (EO-1312C, Edmund optics, Barrington, NJ, USA) connected to the rigid endoscope.

The laparoscopic TC-LTT system consisted of the thermal endoscope, a diode laser (wavelength of 808 nm, BWF2 B&W Tek, Inc, Newark, DE, USA) and a microcontroller (Arduino uno, Arduino, Italy) controlled by a PC (Fig. 5B). The laser beam is guided by an optical fiber (NA 0.22, Ceramoptec, Bonn, Germany) and emitted from the endoscope tip through the endoscope channel. The change in the size of the beam spot at the irradiated site when the distance between the fiber tip and the irradiated site is varied is shown in Suppl. Fig. S5. The temperature information acquired by the infrared thermography sensor is transmitted to the microcontroller. Based on the temperature information, the appropriate laser irradiation output is calculated to keep the temperature of the irradiation target constant.

The laparoscopic TC-LTT system was used with a laparoscopic insufflation device (PNEUMO SURE, Stryker, San Jose, CA, USA) equipped with a light source device (L10000, Stryker, San Jose, CA, USA) (Fig. 5C).

### Operation of the TC-LTT system

The temperature distribution of the area observed by the thermography sensor and the bright field image of the area observed by the CMOS camera were each monitored. On the temperature monitor, the pixel with the highest temperature was displayed as a red dot or green dot: red dot when the laser is on and green dot when the laser is off. In addition, 9 × 9 pixels around the red/green pixel were automatically extracted and four vertices of the square formed by the 9 × 9 pixels were displayed as blue dots (Video 1). Simultaneously, the average of the temperatures of the 81 pixels (9 × 9 pixels) was automatically calculated, and we defined the average temperature as "temperature of the irradiated target".

A surgeon confirmed the location of the tumor on the bright field monitor and advanced the optical fiber via the channel until the fiber tip appeared on the bright field monitor. Next, the tumor was irradiated through the optical fiber in a non-contact manner. Based on the "temperature of the irradiated target", the target tumor was heated with maintenance of temperature by automatic calculation of the appropriate power of the laser irradiation.

During the laser irradiation, the position of the laparoscopic endoscope was manually corrected to ensure that the laser irradiation site did not dislocate significantly from the tumor. When the irradiated area was far from the tumor, the laser output was stopped.

### Methods for evaluating anti-tumor effects

TC-LTT was performed in an orthotopic hepatocellular carcinoma model rat (preparation method described later). The experimental animals were randomly divided into two groups: a treatment group (n = 6) and a control group (n = 7). After induction of general anesthesia, a 15 mm trocar (VersaOne Optical Trocar 15 mm, COVIDIEN, Norwalk, CT, USA) was inserted into the abdominal cavity through a 1.5-cm skin incision. The thermal laparoscopic camera was inserted via the trocar and insufflation with CO_2_ gas (insufflation pressure of 3 mmHg) was performed. For the treatment group, laser irradiation was performed at a temperature setting of 70 °C for 300 s. The beam spot size in this study was assumed to be about 10 mm according to Suppl. Fig. S5. In previous experiments, we confirmed that this heating setting (70 °C for 300 s) has a therapeutic effect on the entire tumor area (Suppl. Fig. S1).

The rats were sacrificed one week after the thermal therapy. Liver lobes were extracted and fixed in 10% formaldehyde solution and then subjected to the process of HE staining. The size of the tumor was measured with a digital caliper at the time of tumor extraction, and the estimated volume was calculated as follows: (length) × (width) × (height) × 1/6π.

### Statistical methods

Statistical analysis was performed using the Mann–Whitney U test. The statistical package used was JMP 14 (SAS Institute Inc., Cary, NC, USA). *P* < 0.05 was considered to be statistically significant.

### Method for preparing orthotopic hepatocellular carcinoma model rats

#### Cell line

Rat hepatocellular carcinoma strain N1-S1 cells (CRL-1604, ATCC, Manassas, VA, USA) were used. The culture medium was Dulbecco's Modified Eagle medium supplemented with 10% FBS, penicillin (100 U/mL) (Thermo Fisher, Waltham, MA, USA), streptomycin (100 µg/mL) (Thermo Fisher, Waltham, MA, USA), and amphotericin B (0.25 µg/mL) (Sigma-Aldrich, St. Louis, MO, USA). The cells were incubated in an incubator at 37 °C in 5% CO_2_ and 95% air.

#### Animals

Female SD rats (Japan SLC, Hamamatsu, Japan) at 8 weeks of age were used in this study. The rats were housed at 3 ~ 4 per cage under controlled temperature (23–25 °C) and relative humidity (50%) with 12 h of light (7:00–19:00). All animal procedures were performed in accordance with the guidelines approved by the National Defense Medical College Animal Care and Use Committee (Permit number: 19009).

#### Establishment of a rat tumor model

SD rats were injected intraperitoneally with a mixture of anesthetics: medetomidine (0.3 mg/kg) (Nippon Zenyaku Kogyo Co., Ltd., Japan), midazolam (4.0 mg/kg) (Sandz Corp., Japan), and butorphanol (5.0 mg/kg) (Meiji Seika Pharma Co., Ltd., Japan). After a small laparotomy, the left lobe of the liver was led out of the body, and 20 µL of a PBS-based cell suspension (3.5 × 10^4^ cells/µL) was injected by puncture with a 30 G needle under the liver capsule. One week after transplantation of the cell suspension, the rats were used as liver tumor model rats.

This study follows the recommendations in the ARRIVE guidelines (https://arriveguidelines.org).

## Supplementary Information

Supplementary Video 1.

Supplementary Information 1.

## Data Availability

The datasets analyzed during the current study are available from the corresponding author upon reasonable request.
