# Supplementary material for: Temperature-controlled laser thermal therapy system using a newly developed laparoscopic system equipped with an ultra-compact thermographic camera
Source: Sci Rep. 2022 Oct 31;12:18287. doi: 10.1038/s41598-022-22908-4 (PMC9622731; doi:10.1038/s41598-022-22908-4)
Supplement: Supplementary file 2 — Supplementary Information 1. [file 41598_2022_22908_MOESM2_ESM.docx]

【Supplementary information】

Temperature-controlled laser thermal therapy system using a newly developed laparoscopic system equipped with an ultra-compact thermographic camera

Manabu Harada^1^, Yuji Morimoto^2*^, Ohara Mutsuki^3^, Jun Ohya^3^, Ken Masamune^4^, Yujiro Itazaki^1^, Takao Sugihara^1^, Hironori Tsujimoto^1^, Yoji Kishi^1^, Hideki Ueno^1^

1. Department of Surgery, National Defense Medical College, Saitama, Japan
2. Department of Physiology, National Defense Medical College, Saitama, Japan
3. Department of Modern Mechanical Engineering, School of Creative Science and Engineering, Waseda University, Tokyo, Japan
4. Faculty of Advanced Techno-Surgery, Institute of Advanced Biomedical Engineering and Science, Tokyo Women’s Medical University, Tokyo, Japan

**1. A study of heating temperature and therapeutic depth**

【Methods】

In order to clarify the relationship between the set temperature of heating and the therapeutic depth, temperature-controlled laser thermal therapy was performed using the orthotopic rat hepatocellular carcinoma model at 2 weeks after transplantation of the cell suspension.

After the rats had been anesthetized, a 5-cm skin incision was made to expose the liver tumor for laser irradiation. Using the temperature-controlled near-infrared irradiation system that we reported previously^1^, near-infrared irradiation (808 nm) was performed for 300 s (maximum power of 3 W/ cm^2^) at each temperature setting (45, 50, 60, 70, 80, and 100°C). The rats were sacrificed at 2 days after the treatment, and the irradiated liver lobes were harvested and fixed in 10% formaldehyde solution. Tissue samples were cut along the laser irradiation axis, and the maximum length of the necrotic area in the HE-stained specimens was measured.

【Results】

The median (interquartile range) necrotic depths of penetration at set temperatures of 45°C, 50°C, 60°C, 70°C, 80°C, and 100°C were 4.1, 5.2, 6.0, 6.4, 9.3, and 7.5 mm, respectively (Suppl. Fig. S1).

Based on the above results, we examined the minimum temperature conditions for fulfilling the conditions of both complete eradication of tumor tissue in the liver cancer model and avoidance of damage to normal liver tissue as much as possible. As a result, the temperature setting was determined to be 70°C in the laparoscopic laser thermotherapy experiment.

【Legend】

Suppl. Fig. S1. Tumor necrosis depth with changing tumor surface temperature (N = 4-5). Up to 80°C, the necrosis depth becomes deeper in a temperature-dependent manner.


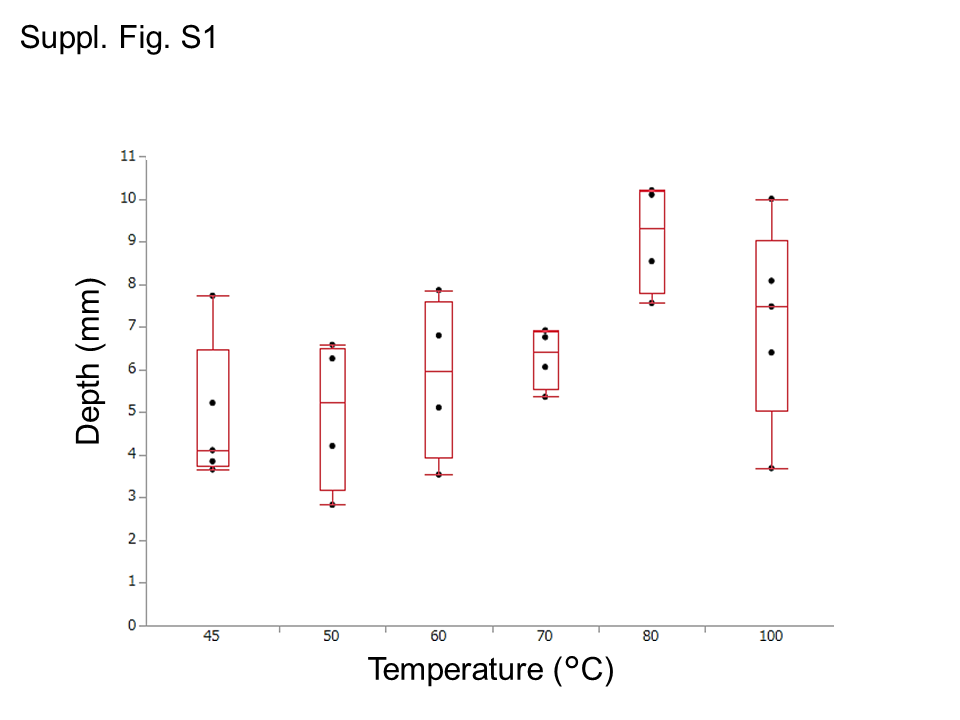


**2. A study of heating time and therapeutic depth**

【Methods】

To clarify the relationship between heating time and therapeutic depth, the therapeutic temperature was set at 70°C and irradiation was performed for 37 s, 75 s, 150 s, or 300 s. The animal model, heating device, and evaluation method were the same as those described in Suppl. Sec. 1.

【Results】

The median (interquartile range) necrotic depths at set times of 37 s, 75 s, 150 s, and 300 s were 5.3, 5.9, 6.5, and 6.4 mm, respectively (Suppl. Fig. S2).

The results of this experiment showed that the necrotic depth obtained with an irradiation time of 300 s can also be obtained with an irradiation time of 150 s. The necrotic depth was smaller in some rats when the irradiation time was 75 or 37 s.

【Legend】

Suppl. Fig. S2. Depths of tumor necrosis with different heating times (N = 4) with temperature set at 70°C.


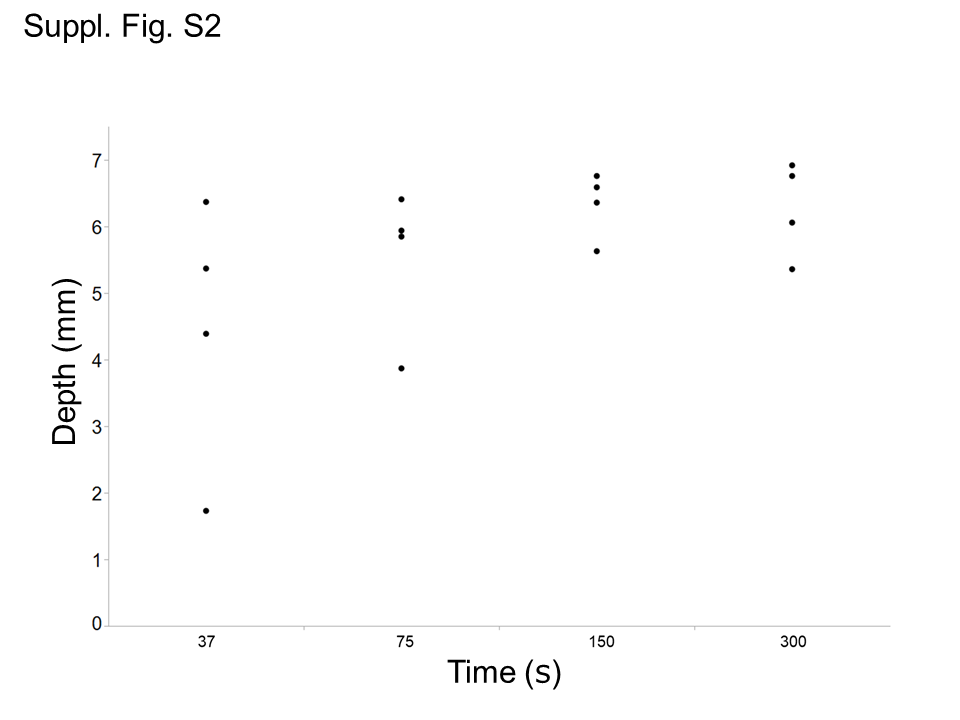


**3. Changes in tissue exposed to laser irradiation without using an automatic temperature control system**

【Methods】

In order to observe tissue changes after laser heat treatment without using an automatic temperature control system, tumors were irradiated with the automatic temperature control system being inactivated, and tumor surface temperatures were measured during laser irradiation.

The animal model and evaluation method were the same as those described in Suppl. Sec. 2.

【Results】

In all cases (N = 3), the temperature monotonically increased with the start of irradiation (Suppl. Fig. S3A). When the temperature exceeded 100°C, carbonization of the tumor surface was observed (Suppl. Fig. S3B). Histopathological examination showed black color changes in the area consistent with carbonization (Suppl. Fig. S3C and S3D).

【Legend】

Suppl. Fig. S3. (A) Tumor surface temperature during laser irradiation without using the automatic temperature control system (N = 3). In all rats, tumor surface temperatures exceeded 100°C. (B) Photograph of the surface of a tumor immediately after the end of irradiation. A blackened area of a few millimeters in size can be seen in the center of the irradiated field, indicating carbonization. (C, D) Histopathologically, a layer of charring in the underlying layer was observed. Photograph D is an enlarged view of the red frame in photograph C. HE, Scale bar = 2.5 mm (C), 0.25 mm (D).

**
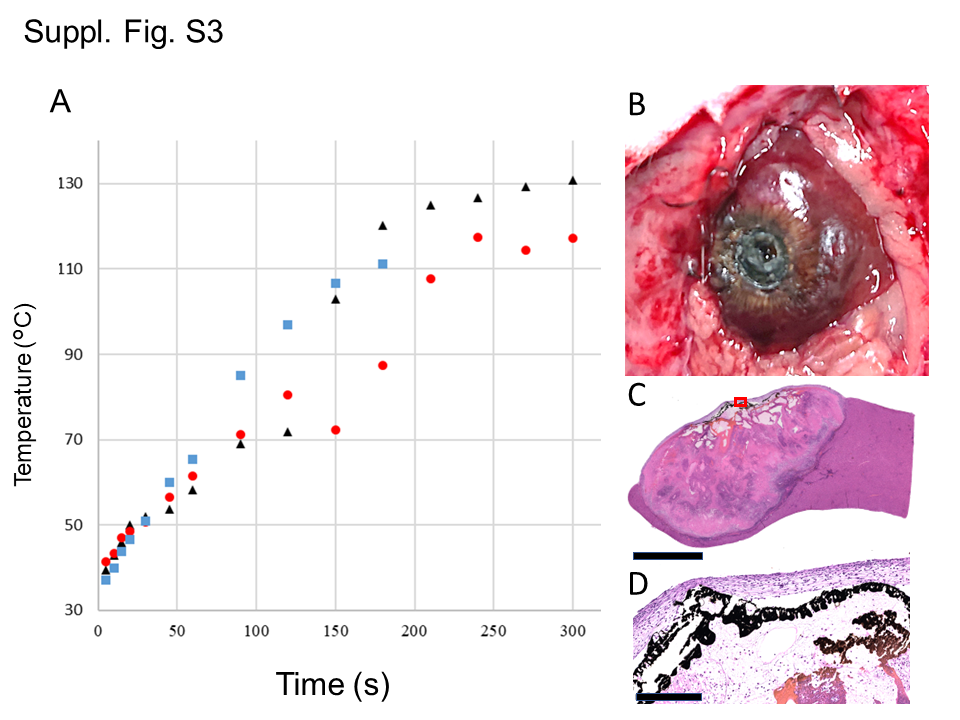
**

**4. Pathological examination of blackened areas in laser-irradiated tissue under control of the TC-LTT system**

【Methods】

Histopathological examination was performed to examine the blackened areas that were sometimes seen in the laser-irradiated tissue when using the TC-LTT system.

Tumors were irradiated at a set temperature of 70°C for 300 s. The animal model and evaluation method were the same as those described in Suppl. Sec. 2.

【Results】

Although the tumor surface temperature during laser irradiation was maintained constant at 70°C (Suppl. Fig. S4B), blackened areas in the surface of hepatocellular carcinoma were seen immediately after the end of laser irradiation (Suppl. Fig. 4A). HE-stained tissue showed no blackened areas, but hemorrhage and congestion were observed on the irradiated surface (Suppl. Fig. S4C and S4D). The blackening is probably due to heat-induced conversion of hemoglobin contained in red blood cells to methemoglobin. This is because hemoglobin has been reported to change to methemoglobin (color: deep brown) with heat^2^.

【Legend】

Suppl. Fig. S4. (A) Photograph of hepatocellular carcinoma after laser irradiation. A blackened area consistent with the irradiated field can be seen. (B) Temperature of the tumor surface over time during laser thermal therapy. The tumor surface was heated at a constant temperature of 70°C. (C) Specimen harvested immediately after irradiation. No carbonization was observed. (D) An enlarged view of the red frame in (C). Hemorrhage and congestion are seen on the irradiated surface. HE, Scale bar = 5 mm (C), 0.1 mm (D).


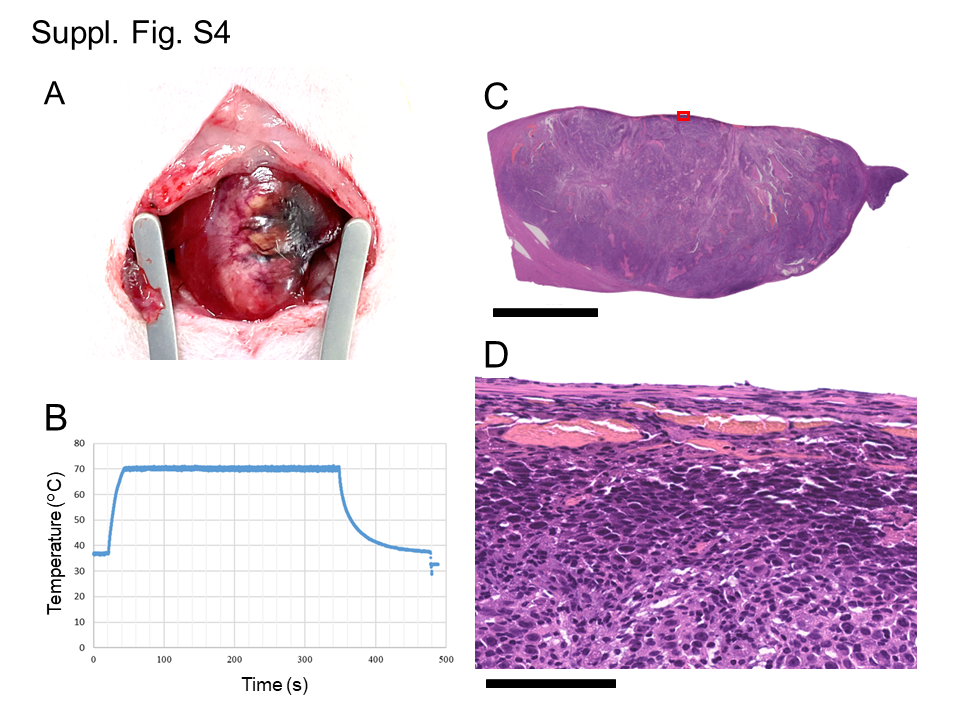


**5. Measurement of beam spot size and estimated fluence rate on the tumor surface**

【Methods】

The beam spot diameter at the irradiated surface was measured with respect to the distance between the optical fiber tip position and the irradiated surface.

An optical fiber (NA 0.22, Ceramoptec, Bonn, Germany) was placed right over the surface of a thermal sensor film (TDCF-510H, LxRay, Tokyo, Japan). With the distance between the optical fiber tip position and the surface of the film being varied, the diameter of the region where the color tone changes (the region where the temperature rises due to laser irradiation) on the film was measured with a digital caliper.

【Results】

The relationship between the distance and beam spot diameter was a regression line with a correlation coefficient (r) of 0.991.

Since the distance between the endoscope tip and the tumor surface was estimated to be about 10 mm during the operation in the rat abdominal cavity, the estimated beam spot diameter on the tumor at that time was about 10 mm (in terms of area, 0.79 cm^2^) as shown in Suppl. Fig. S5. Therefore, the fluence rate during intraperitoneal manipulation can be estimated to be 3.8 W/ cm^2^ at the laser power of 3 W/ cm^2^.

【Legends】

Suppl. Fig. S5. Beam spot diameter with respect to the distance between the optical fiber tip position and the irradiated surface.


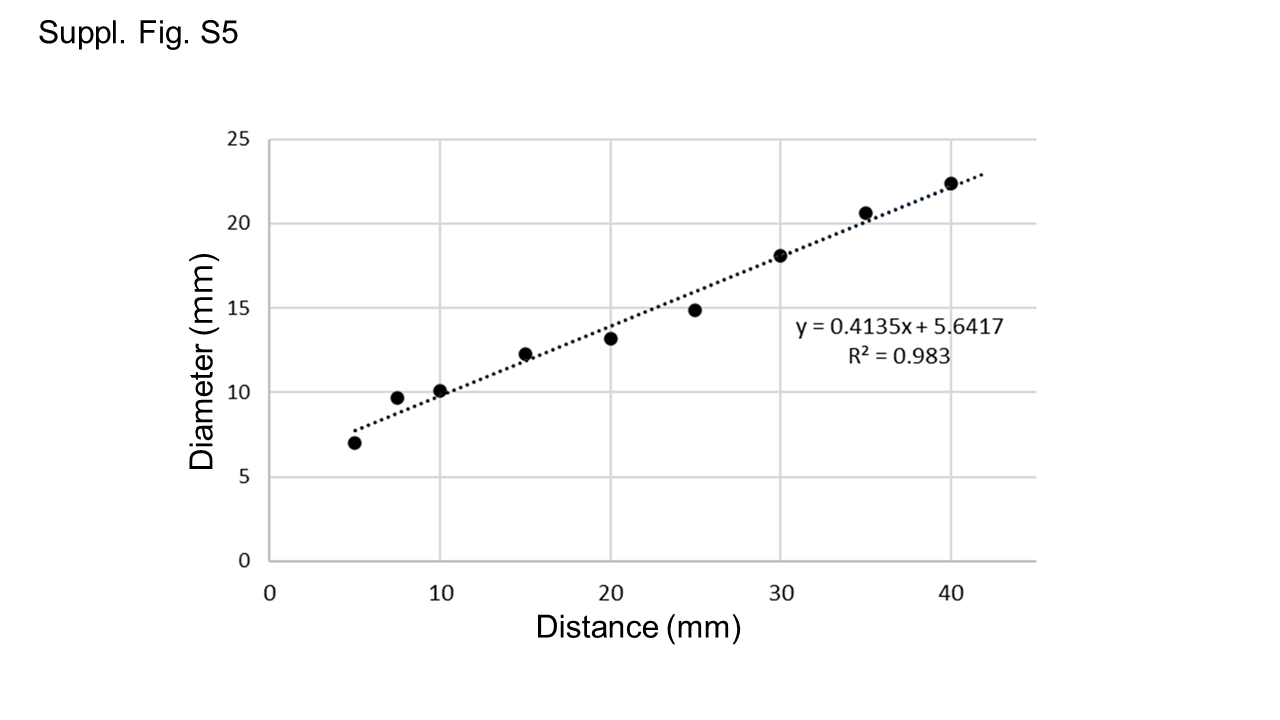


**6. 【Legends : Suppl. Video】**

Video 1. A movie of laser thermal therapy being performed by the thermal endoscope laparoscopically. In the thermal imaging (*left side*), the highest temperature pixel (*red dot*) surrounded by 9 x 9 pixels with the four vertices of the square indicated by *blue dots* can be seen. The highest temperature pixel is indicated by a *green dot* when the laser is off, and the color changes to a *red dot* when the laser irradiation starts. The bright field image (right side) taken with the CMOS camera shows a laser fiber at the 2 o'clock position on the screen (edited at 20x speed).

References

1. Nomura, S. *et al.* Thermal Sensor Circuit Using Thermography for Temperature-Controlled Laser Hyperthermia. *Journal of Sensors*. **2017**: 10.1155/2017/3738046 (2017).

2. Bastide, B., Porter, G. & Renshaw, A. The effects of heat on the physical and spectral properties of bloodstains at arson scenes. *Forensic Sci. Int.* **325**, 110891; 10.1016/j.forsciint.2021.110891 (2021).
